# Correction: Identification and biochemical characterisation of Asp t 36, a new fungal allergen from *Aspergillus terreus*

**DOI:** 10.1016/j.jbc.2021.100379

**Published:** 2021-03-03

**Authors:** Bijoya Karmakar, Bodhisattwa Saha, Kuladip Jana, Swati Gupta Bhattacharya

VOLUME 295 (2020) PAGES 17852–17864

As this particular abbreviation is a fungal allergen name (Asp t 36) given by the WHO/IUIS, it should not be included in the abbreviation list of the manuscript.

